# Bayesian Model Selection with Network Based Diffusion Analysis

**DOI:** 10.3389/fpsyg.2016.00409

**Published:** 2016-04-05

**Authors:** Andrew Whalen, William J. E. Hoppitt

**Affiliations:** ^1^School of Biology, University of St. AndrewsSt. Andrews, UK; ^2^School of Biology, University of LeedsLeeds, UK

**Keywords:** Network Based Diffusion Analysis, Bayesian model selection, WAIC, social learning, statistical methods

## Abstract

A number of recent studies have used Network Based Diffusion Analysis (NBDA) to detect the role of social transmission in the spread of a novel behavior through a population. In this paper we present a unified framework for performing NBDA in a Bayesian setting, and demonstrate how the Watanabe Akaike Information Criteria (WAIC) can be used for model selection. We present a specific example of applying this method to Time to Acquisition Diffusion Analysis (TADA). To examine the robustness of this technique, we performed a large scale simulation study and found that NBDA using WAIC could recover the correct model of social transmission under a wide range of cases, including under the presence of random effects, individual level variables, and alternative models of social transmission. This work suggests that NBDA is an effective and widely applicable tool for uncovering whether social transmission underpins the spread of a novel behavior, and may still provide accurate results even when key model assumptions are relaxed.

## 1. Introduction

There has been a substantial interest in better understanding how and why animals use social information (Heyes, 1994; Laland, 2004; Galef and Laland, 2005), and particularly understanding if certain behaviors diffuse through populations as a result of social transmission (learning from others) (Reader, 2004). A capacity for social transmission has been demonstrated in many species using a traditional demonstrator-observer paradigm (Hoppitt and Laland, 2013). In contrast, recent studies have focused on studying the diffusion of behavior in freely interacting groups of animals in the field (e.g., Allen et al., 2013; Hobaiter et al., 2014) or in the laboratory (e.g., Boogert et al., 2008; Atton et al., 2012), aiming to asses the importance of social transmission in the spread of behavior, and elucidate typical pathways of transmission. However, in many cases it can be challenging to determine whether the spread of behaviors is facilitated by social transmission, or purely the product of independent asocial learning. This challenge increases the difficulty in inferring the presence of, and understanding the mechanisms that underlie social learning in animals.

An early approach in detecting social transmission and asocial learning was to analyze the shape of the “diffusion curve,” the number of animals in the population who had performed the novel behavior over time (Lefebvre, 2000). The theory was that if the diffusion followed an accelerating pattern, or an “s-shaped curve,” this was likely a product of social transmission (Reader, 2004). However “s-shaped curves” can also be produced by other mechanisms, like individual differences in the rates of learning, which has lead to the technique to be considered unreliable (Reader, 2004; Franz and Nunn, 2009; Hoppitt et al., 2010).

More recent research has responded to concerns over the validity of diffusion curve analysis to develop novel statistical tools to analyze the rates of diffusion of novel behaviors. Network Based Diffusion Analysis (NBDA), is one such approach that infers social transmission if the spread of the novel behavior follows a social network (Franz and Nunn, 2009; Hoppitt et al., 2010). In most cases the social network is a pre-established association network (e.g., Aplin et al., 2012; Allen et al., 2013) that is assumed to reflect opportunities for learning between each pair of individuals (Hoppitt et al., 2010). However, the networks used can instead directly reflect the pattern of recorded (probable) observations among individuals if such information is available (Hobaiter et al., 2014), or different networks can be used to represent different hypotheses about the pathways of transmission (e.g., Farine et al., 2015). In addition to analyzing the diffusion of behavior in natural populations, NBDA has also been used in laboratory studies (e.g., Atton et al., 2012). Here, fewer individuals are used, but with the advantage that replicate groups can be easily created, and researchers can track the diffusion of multiple behavioral traits through the same groups (e.g., Boogert et al., 2008). NBDA then potentially allows researchers to make inferences about individual differences in innovation and social learning ability. As such NBDA has potential to provide a unifying analytical framework for studying social transmission in the laboratory and the field.

Although initially a frequentist method, NBDA has been recast into a Bayesian framework to allow better specification of different models of social learning, and the inclusion of random effects allowing for correlations in learning rate within individuals when they are subjected to multiple diffusions (e.g., Boogert et al., 2014; Nightingale et al., 2014). Although the move to a Bayesian model of NBDA has advantages, one of the disadvantages has been the ability for researchers unfamiliar with the method to use it, a problem exacerbated by the existence of a number of alternatives for model selection, several of which have been used in the context of NBDA.

In this paper, we address these issues by presenting and evaluating a framework for performing NBDA where model selection is done using the Watanabe Akaike Information Criteria (WAIC; Watanabe, 2013). Use of WAIC has the advantage that it is computationally relatively more straightforward to implement than alternatives like reversible jump Markov chain Monte Carlo (RJMCMC). One of the goals of this paper is to evaluate the performance of WAIC in performing model selection in the context of NBDA, and use a large simulation study to examine how the performance changes if key model assumptions are not met.

## 2. Network based diffusion analysis

NBDA is a general framework for evaluating different hypotheses for the spread of a novel behavior. At its core, NBDA relies on a two-step process to evaluate, and select a most likely model that describes observed data. First, we construct a likelihood function to represent the likelihood that each model generated the observed data. Many of these likelihood functions require the values for large number of parameters to be estimated. To estimate these parameters, Bayes' rule is used to fit the parameters to the data. The resulting model and parameters are then assessed using WAIC, which evaluates the predictive fit of each model. In the simplest case the model with the best (lowest) WAIC is chosen as our model.

NBDA falls under a wide class of hierarchical Bayesian models (Gelman et al., 2014), and many of the steps below are applicable to a broad range of settings. NBDA is distinguished from other hierarchical models by explicitly modeling social influences on learning. There are two variants of NBDA. Time of Acquisition Diffusion Analysis (TADA) analyzes the time at which an animal first performs a novel behavior, and can be analyzed in continuous (Hoppitt et al., 2010) or discrete (Franz and Nunn, 2009) time. In contrast, Order of Acquisition Diffusion Analysis (OADA) analyzes only the order in which animals first perform the behavior. Here we focus on continuous TADA and evaluate the effectiveness of NBDA with WAIC in this context, although the same approach is applicable to other variants of NBDA like OADA.

## 3. Time of acquisition diffusion analysis

Time of Acquisition Diffusion Analysis is a modeling technique which evaluates whether the rate at which an individual first perform a novel behavior is dependent on the behaviors of other individuals in that population. Because the method focuses primarily on acquisition of a novel behavior, it typically only analyzes the initial performance of the behavior and ignores subsequent performance.

As an example, imagine a population of birds learning to flip open the lid of container to receive a food reward. When the task is initially presented, none of the birds are able to solve it. Over a long period of time, all or most of the birds are eventually able to solve the task and receive the food reward. The question we wish to ask is, was the spread of the novel behavior (flipping the lid) acquired through asocial learning alone, or asocial learning aided by social transmission. If learning was done through pure asocial learning (including the effects of individual-level covariates), then the rate at which each bird solves the task should be constant (though this assumption can be relaxed Hoppitt et al., 2010), and independent of other birds having solved the task. If the learning was aided by social transmission, that rate of solving should increase as more other birds solve the task.

We can formalize this logic using an instantaneous rate model. This model assumes that at each instant, a given bird has some chance of learning the novel behavior. In the case of asocial learning, this rate does not depend on the number of other birds that had previously solved the task. In the case of social transmission, this rate will be sensitive to other birds solving the task.

To generate a likelihood function, we assume that at each instant the rate that bird *i*, solves the task (i.e., acquires the novel behavior) is λ_*i*_(*t*), where

(1) $$\begin{array}{l} \lambda_{i}\left(t\right)=a_{i}\left(t\right)+s_{i}\left(t\right), \end{array}$$

the sum of an asocial learning rate *a* and social learning rate *s*. These rates are allowed to change over time, allowing the model to capture changes in the birds' environment, and the birds' social environment.

There may be individual-level differences in the rate at which the birds learn novel behaviors, to capture these differences we parameterize asocial learning as

(2) $$\begin{array}{l} a_{i}\left(t\right)=exp\left(\lambda_{0}+A_{i}+\phi_{i}\right), \end{array}$$

where λ_0_ stands for a base rate of learning, modified by some set of individual-level covariates, *A*_*i*_ and individual-level random effects ϕ_*i*_.

To incorporate social information we must provide a model of how an individual's learning rate is influenced by other animal's actions. In TADA we assume that animals are only influenced by the number of other individuals who have solved the task. We assume that the social learning rate is

(3) $$\begin{array}{l} s_{i}\left(t\right)=\sigma_{i}S_{i}\left(t\right), \end{array}$$

where σ_*i*_ is an individual-level rate which determines the influence of social information and *S*_*i*_ captures how much social information is in the environment. Like asocial learning, we allow for individual-level differences in social learning ability,

(4) $$\begin{array}{l} \sigma_{i}\left(t\right)=exp\left(s_{0}+B_{i}+\psi_{i}\right), \end{array}$$

where *s*_0_ is a base rate of social learning, *B*_*i*_ the influence of individual-level covariates on social learning, and ψ_*i*_ the influence of individual-level random effects on social learning. *S*_*i*_ is given by

(5) $$\begin{array}{l} S_{i}\left(t\right)=\sum a_{ij}I_{i}\left(t\right), \end{array}$$

where *a*_*ij*_(*t*) is the influence individual *i* has on individual *j*, and *I*_*j*_(*t*) is an indicator variable that is 1 if animal *j* has solved the task prior to time *t*, and 0 otherwise. The amount of influence each individual exerts on each other can be captured by a social network which can be empirically estimated (Boogert et al., 2008). If there are no network differences, then *S*_*i*_(*t*) = ∑*I*_*j*_(*t*) or the number of animals who have solved the task at each point in time. This model assumes that the rate of learning due to social transmission depends linearly on the number of other animals who have solved the task. In reality, other learning rules are possible, and we discuss some of these below.

In TADA, the rates of solving are estimates from the observed data using a hazard model, where if an individual has not solved the task at time *t*_0_ then the likelihood that they solve the task at *t*_1_ is,

(6) $$p(s_{i}=t)=\lambda_{i}(t_{1})\int_{t_{0}}^{t_{1}}exp(\lambda_{i}(t))dt,$$

and the likelihood that they fail to solve the task by time *t*_1_ is

(7) $$p(s_{i}>t_{1})=\int_{t_{0}}^{t_{1}}exp(\lambda_{i}(t))dt.$$

Note that the parametrization of the model presented here differs from the original presentation of the continuous TADA (Hoppitt et al., 2010), although it follows that used for previous versions of Bayesian NBDA (Nightingale et al., 2014). In the original formulation, a parameter λ_0_ = *exp*(λ_0_) gives the baseline rate of asocial learning, and a parameter *s* = *exp*(*s*_*o*_)∕*exp*(λ_0_) gives the rate of social transmission per unit connection, relative to the baseline rate of asocial learning. The parametrization presented is better suited for Monte Carlo sampling (Nightingale et al., 2014) but estimates for the parameter for the original specification can still be obtained from the posterior distribution.

Using the hazard model and our parametrization of the learning rate, we can estimate the rates of different model parameters based on a given data set by using Bayes' rule. Bayesian inference can be accomplished by a multi-functional statistical software packages like JAGS or Stan. In this study, we use a hand-coded Monte Carlo sampler implemented in R (R Core Team, 2013). Once posterior distributions are obtained, we can then use a model selection technique to compare between different models of learning. We outline one model selection approach below.

## 4. Model selection using WAIC

The goal of model selection is to compare multiple competing models, given a data set, and determine a single, or set of likely candidate model(s) that are thought to be “best.” In the context of NBDA, a primary goal of the model selection is often to tell if a social model describes the data better than a purely asocial model. Although there are many ways of defining a best model, in this context, we evaluate models based on their predictive validity. Other approaches are discussed in the discussion.

Predictive validity is an assessment of the ability of each model to predict the results of future experiments, or unanalyzed tasks. However, true measures of predictive validity can be hard to obtain since it is often not feasible, or expensive to collect further data. A traditional alternative is to examine the performance of the model on predicting already obtained data. In the best case, “leave one out” cross validation trains the model on all but one piece of data, and then examines how well the model predicts the left out piece of data. This process is repeated for every data point. This technique is computationally expensive with a large number of data points, and may be inappropriate with few data points, since leaving a single data point out may be a large fraction of the total data.

There exists alternatives to leave one out cross validation like information criteria, a set of techniques (including WAIC) for balancing goodness of fit to collected data, against the number of parameters in the model. In some cases these techniques are asymptotically equivalent to leave one out cross validation as the number of data points grows large, while being much more computationally tractable to compute.

In our case, we use the WAIC to score each model. WAIC is a new alternative to older information criteria like Akaike's Information Criterion (AIC; Akaike, 1973) or Deviance Information Criterion (DIC; Spiegelhalter et al., 2002). It has a number of advantages over AIC and DIC. Unlike AIC or DIC, which assess a model's fit based on a single point estimate, WAIC uses the entire Bayesian posterior distribution, making it more accurate when the posterior distribution is not normally distributed. The results of WAIC also asymptotically approach Bayesian cross validation in the large sample limit (Watanabe, 2013).

WAIC assesses model fit by computing the ability of each model to predict the entire data set that it is fit on, penalizing models that have an un-even fit across individual pieces of data. WAIC can be calculated by Gelman et al. (2014):

(8) $$\begin{array}{l} WAIC=-2\left(lppd-p_{WAIC}\right), \end{array}$$

where *lppd* is the logpoint-wise predictive density, and pWAIC is a term that penalizes models with large numbers of parameters. The factor of −2 brings WAIC to be on the same scale as other information criteria. lppd is approximated using the posterior output of an MCMC chain by:

(9) $$\begin{array}{l} lppd=\sum log\left(E_{post}p\left(y_{i}|\theta \right)\right), \end{array}$$

where the outer sum is over individual data points, and the inner term is the expectation of the likelihood over the entire posterior sample. This value is then corrected for the estimated number of parameters of the model by

(10) $$\begin{array}{l} p_{WAIC}=\sum var_{post}log\left(p\left(y_{i}|\theta \right)\right), \end{array}$$

where the inner term is the variance of the likelihood over the entire posterior sample for each data point. This remaining term penalizes models that have uneven (i.e., high variance) fit across different data points, which may be an indication of over fitting.

To perform model selection, we can fit multiple potential models to the data and use WAIC to evaluate their predictive value, selecting the model with the smallest WAIC value as our chosen model.

For example, if the question of interest is whether or not animals use social transmission to acquire behaviors, we could fit two models, one where animals are assumed to use social information, and a second where they are assumed to only use asocial information. After fitting both models and calculating the WAIC for them we can assess which model has a better fit, and whether the animals are more likely to have used social information, or to have relied only on asocial learning.

One open question is how big does the WAIC difference between models need to be, in order to be considered indicative of a true preference for one model. There is no hard and fast rule, although the difference should be greater than the variation due to Monte-Carlo sampling often greater than 1, or in the case study presented below, greater than 5.

## 5. Simulated performance of NBDA

To evaluate the expected reliability of NBDA, we performed a large-scale simulation study to understand under what conditions NBDA will accurately determine whether social or asocial information was being used to solve the task. In this study, we used a single model to generate the results of 1000 diffusion experiments, and used NBDA to infer the model used to generate each simulation. The accuracy of NBDA is then the likelihood that the model inferred by NBDA was the model used to generate the data.

Unless otherwise noted, each simulation followed the same design. We considered a population of ten animals who were given a novel foraging task (similar to Boogert et al., 2008, 2014). In the task each animal was required to learn how to get a piece of food out of a container (e.g., by removing a lid). As each animal solved the task (got the food out of the container) the container was replenished giving the other animals a chance to solve the task. Each experiment was run until each animal in the group had “solved” the novel task at least once. This process was repeated ten times with new foraging tasks with the same group of animals. Although we consider primarily novel foraging behaviors here, the framework presented above, and the results presented below, likely will also hold for animals learning a range of new behaviors.

The data for each experiment was generated by turning the hazard model above, into a generative model. For each simulations a model of learning was chosen (e.g., asocial learning without random effects, social learning with random effects), and parameter values for the model were drawn from our prior distribution (see Table 2). The models, including parameters and the distributions for each parameter are given in Tables 1, 2. The distributions given in Table 2 were also used as the prior distribution for performing Bayesian inference (see below). Individual-level effects were treated as a product of an underlying measurable property, η, and a rate term *a* or *b*. We assumed that η was normally distributed with mean 0.

**Table 1:** **Model parameters and the rate equation for each model used in the simulation study**.

**Model name**	**Parameters**	**Rate equation**
Asocial	λ_0_	λ = *exp*(λ_0_)
Social	λ_0_, *s*_0_	λ = *exp*(λ_0_) + *exp*(*s*_0_)*S*
Asocial with random effects	λ_0_, ϕ	λ = *exp*(λ_0_ + ϕ_*i*_) + *exp*(*s*_0_)*S*
Social with random effects	λ_0_, *s*_0_, ψ	λ = *exp*(λ_0_) + *exp*(*s*_0_ + ψ_*i*_)*S*
Asocial with individual-level effects	λ, *A*	λ = *exp*(λ_0_ + *A*_*i*_)
Social with individual-level effects	λ_0_, *s*_0_, *B*	λ = *exp*(λ_0_) + *exp*(*s*_0_ + *B*_*i*_)*S*
Linear social model	λ_0_, *s*_0_, ϕ, ψ	λ = *exp*(λ_0_ + ϕ_*i*_) + *exp*(*s*_0_ + ψ_*i*_)*S*
Diminishing returns social model	λ_0_, *s*_0_, ϕ, ψ	$\lambda =exp\left(\lambda_{0}+\phi_{i}\right)+exp\left(s_{0}+\psi_{i}\right)\sqrt{S}$
Threshold social model	λ_0_, *s*_0_, ϕ, ψ	λ = *exp*(λ_0_ + ϕ_*i*_) + *exp*(*s*_0_ + ψ_*i*_)*sign*(*S*)

*The parameters refer to the inclusion of the terms in the model described in Equations (1)–(5). λ_0_ and s_0_ are the baseline asocial and social learning rates, A and B represent inclusion of a individual level effect on asocial or social learning, and ϕ and ψ are random effects for asocial and social learning*.

**Table 2:** **Distribution of model parameters for the simulation study**.

**Parameter**	**Distribution**
λ_0_	Uniform(−7, −5)
*s*_0_	Uniform(−7, −5)
ϕ_*i*_	Normal(0, 1)
ψ_*i*_	Normal(0, 1)
*a*	Uniform(−1, 1)
*b*	Uniform(−1, 1)

*These parameter distributions were also used as prior distributions for performing inference on each simulation*.

We allowed individuals to use the social information provided on either a homogeneous network, or on a lesioned network. As in Equation (5), we defined a social network to be an association matrix A, whose elements *a*_*ij*_ represent the amount of influence (between 0 and 1) that individual *i* has over individual *j*. In the homogeneous network, all individuals had the same influence over each other; *a*_*ij*_ = 1 for all *i* and *j*. In the lesioned network, the network was initialized the same way as the homogeneous network and then half of the network connections were removed, (*a*_*ij*_ = 0. Because there may be cases where researchers are not able to measure the network accurately, we examined three alternative, inaccurate social networks, which incorrectly assessed the value of 25, 50, or 75% of the network connections—setting a connection to 1 if it was 0, or to 0 if it was previously 1.

We considered three models of social learning. In all cases, we assumed that the social information was transmitted on a homogeneous network. In the *Linear* model, social information term was set to *s* = *ck*, where *k* is the number of animals who have already solved the task (*c* is a constant). In the *Diminishing Returns* model, social information was set to $s=c\sqrt{k}$, to model the fact that subsequent solvers may have a diminishing influence. The choice of the square-root function to model this process was arbitrary; preliminary simulations suggest that similarly shaped functions produce analogous results. In the *Threshold model*, social information was set to *s* = *cI* where *I* is 1 if at least one other individual has solved the task, and 0 otherwise.

After each simulated experiment, NBDA was performed using TADA, with the assumption that there was no social network between individuals. We used WAIC to evaluate a number of alternative models and select the best model. Unless otherwise stated, we ran 1000 simulated experiments for each set of models, with different parameter values for each experiment.

In the first set of simulations we look at recovering the correct underlying model and parameter values for when learners use only asocial learning (with random effects), or asocial learning and social transmission (with random effects). In the next set of simulations we examined whether we could correctly infer the influence of individual-level variables on learning. In the last set of simulations, we also examined whether we could correctly infer the underlying model of social learning for each experiment.

### 5.1. Model and parameter recovery

**Figure 1:**
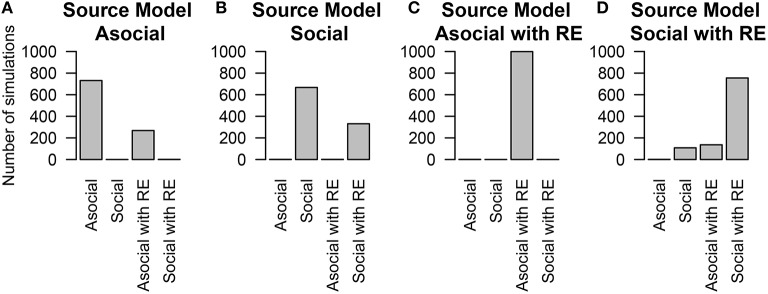
**The number of simulations each model was inferred to have been the source of the given data out of 1000 total simulations**. The data was either generated from an **(A)** asocial model, **(B)** social model, **(C)** asocial model with random effects (RE), or **(D)** a social model with individual random effects (RE).

To estimate our ability to recover the correct underlying model used to generate the data set, we simulated diffusion based on four models: the asocial, social, asocial with random effects, and social with random effects models. The performance of model recovery is given in Figure 1. Overall the statistical technique was able to determine whether or not a model uses asocial information or social information, but there was a high false positive rate for detecting the presence of random effects, shown by the large number of asocial models without random effects being inferred to be asocial models with random effects (Figure 1A), with the same holding true for social models (Figure 1B). However, even though this method occasionally infers the presence of random effects it is still able to distinguish between social and asocial models, e.g., data generated from a social model may be inferred to have been generated from a social model with random effects, but is unlikely to be inferred to have been generated from either an asocial model, or an asocial model with random effects. This suggests that it may appear that there is underlying variation in social and asocial learning ability, where no such variation exists.

**Figure 2:**
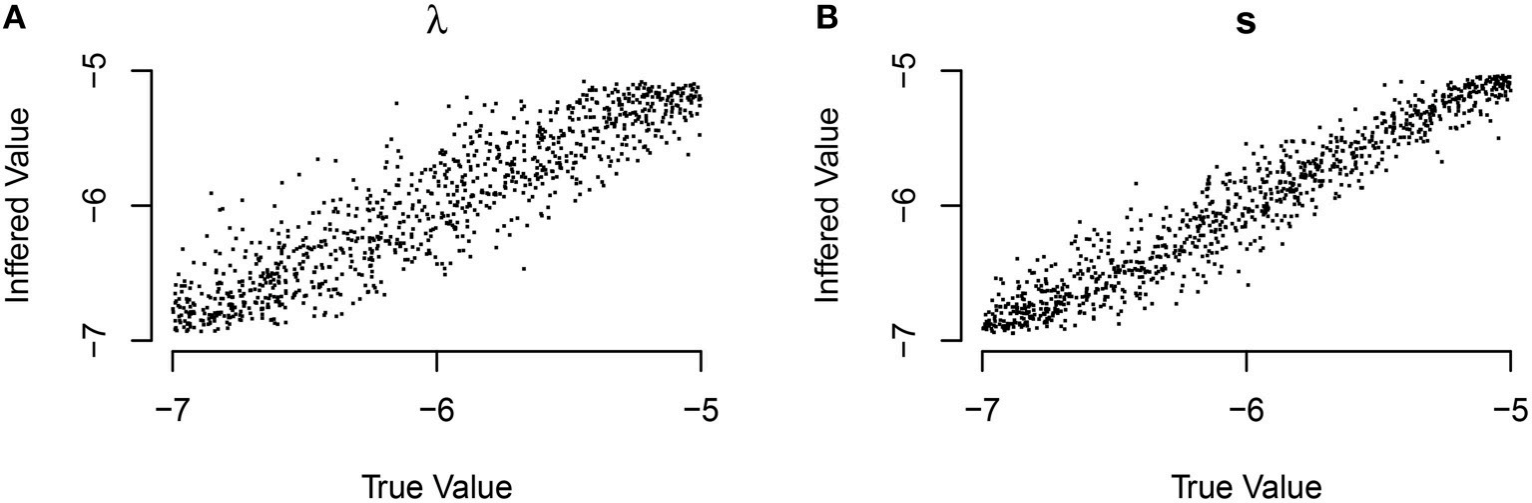
**Inferred model parameters compared to the true values of those parameters for the (A) asocial (λ) and (B) social (S) models**. Perfect parameter recovery would be indicated by a straight line.

We are also interested in whether or not we can correctly infer the underlying learning rates. We assess this question by looking at the social learning model without random effects. The inferred (median) asocial learning and social transmission rates compared to the true rates are given in Figure 2.

The performance of inferring the correct value of the random effects was substantially lower, although in most cases this method correctly inferred the relative ordering of the random effect values (i.e., which bird learned faster) but mis-estimated the absolute value of the random effect values. The technique's ability to recover the ordering (expressed by the within-experiment correlation between the inferred values and the true values) is fairly good, with over 85% of the time the Spearman correlation coefficient was higher than 0.9.

These findings suggest that researchers are safe in using NBDA to infer population average rates of asocial learning, and social transmission, and thus draw conclusions about the overall importance of each (the primary goal of NBDA), if the model is correctly specified (see below). However, we suggest that researchers should not take estimates of individual variability in asocial learning and social transmission too seriously, but are safe to use the technique to obtain rankings of individual abilities in these domains.

### 5.2. Individual-level effects

**Figure 3:**
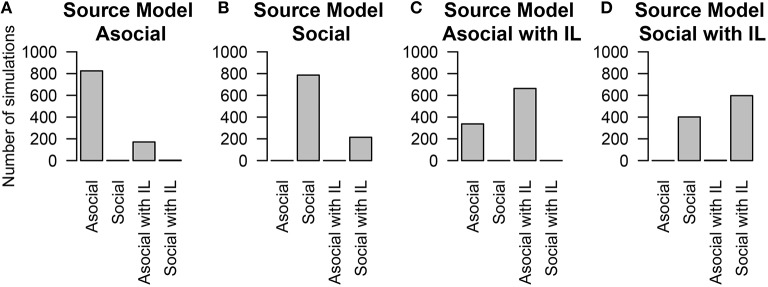
**The number of simulations each model was inferred to have been the source of the given data out of 1000 total simulations**. The data was either generated from an **(A)** asocial model, **(B)** social model, **(C)** asocial model with individual level effects (IL), or **(D)** a social model with individual level effects (IL).

There has been recent interest in understanding which other traits that an individual possesses might correlate with asocial or social learning abilities (e.g., Boogert et al., 2006, 2008). Individual-level effects allow us to include the influence of these covariates in our model. We found that NBDA could correctly interpret the presence of correlates for social, or asocial information some of the time. We also found that the technique could reliably estimate the true value of the covariate; and find that for the individual-level asocial effects 94.7% of the time the true value is within our 95% likely interval, and for the social effects 95.3% of the time the true value is within our 95% likely interval. We do find a high false negative rate in inferring the presence of a covariate (see Figure 3; although this technique was able to distinguish between models that included social learning and those that did not).

These results may in part be due to the presence of covariates that have only a weak impact on learning. In the simulations the influence of the covariates varied between −1 and 1, meaning that some of the time, they could have a very small influence on social learning ability i.e., the parameter can be close to 0. We explored how the impact size of the covariate determined the likelihood of determining the correct model. We found that if the parameter had a large impact, most of the time the technique could recover its presence. However, if the parameter had a small impact, the technique had a high false negative rate.

Therefore, we advise that instead of just attempting to infer whether an individual-level effect is present or absent, researchers use the posterior distribution for a parameter to give credible intervals for how big or small the effect might be. Where these intervals are sufficiently small, conclusions can be drawn about the importance or lack of importance of that effect (Nakagawa and Cuthill, 2007).

### 5.3. Inaccurate social networks

We examined how NBDA would perform when diffusions followed a social network, and when researchers had incorrect knowledge of the social network. In these simulations, individuals had a baseline asocial and social learning rates, and their learning followed a lesioned social network.

**Figure 4:**
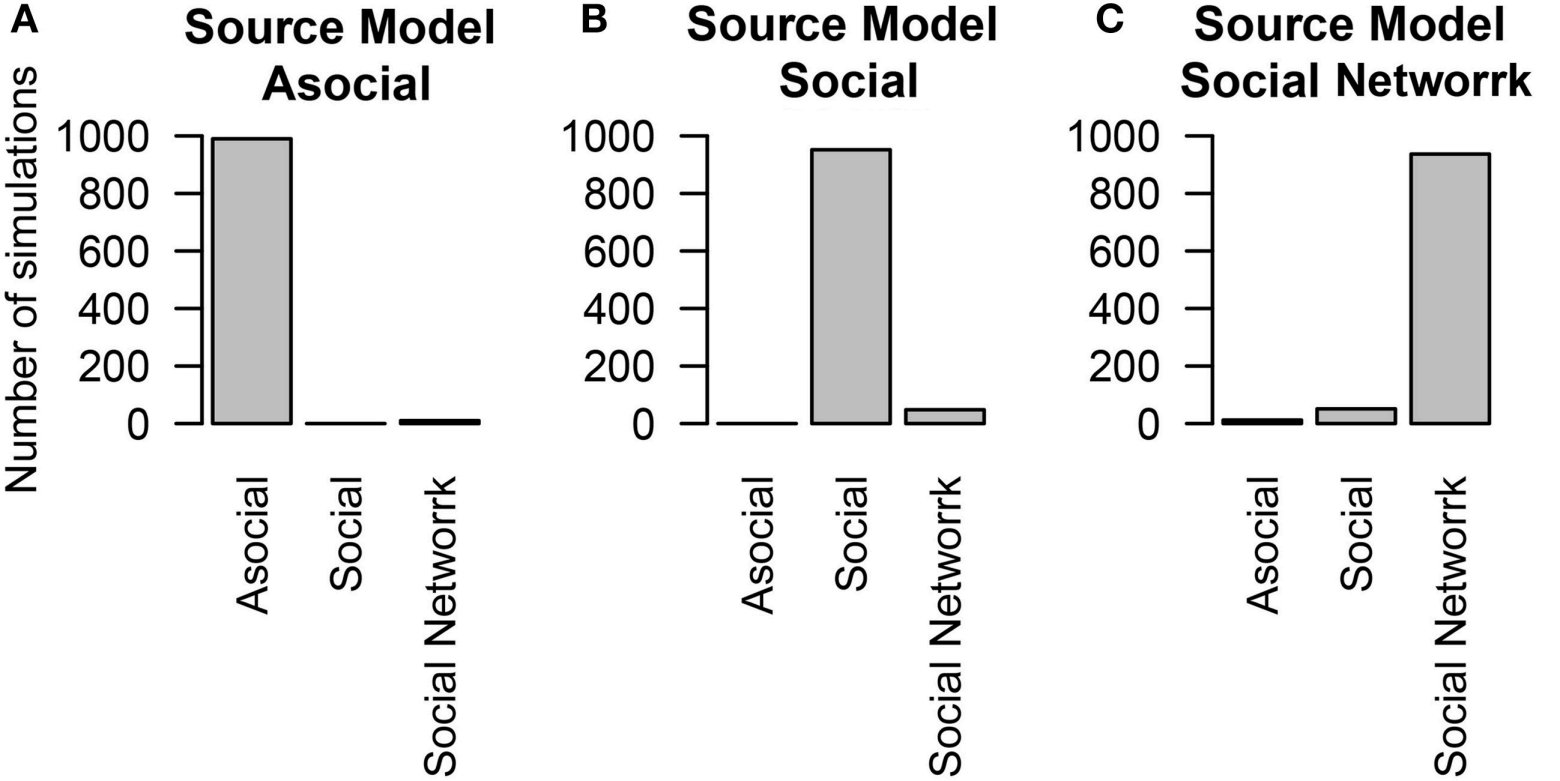
**The number of simulations each model was inferred to have been the source of the given data out of 1000 total simulations**. The data was either generated from different models of diffusion across a social network, either through an **(A)** asocial model, **(B)** social model (i.e., homogeneous network), **(C)** social model with a non-trivial social network. Details of the social network structure can be found in Section 5.

We found that even when social learning followed an association network, the technique was able to determine the influence of social learning (Figures 4B,C) and of asocial learning (Figure 4A). The ability to distinguish between social and asocial learning was not substantially reduced when no knowledge of the network was known and it was assumed that transmission followed a homogeneous network.

**Figure 5:**
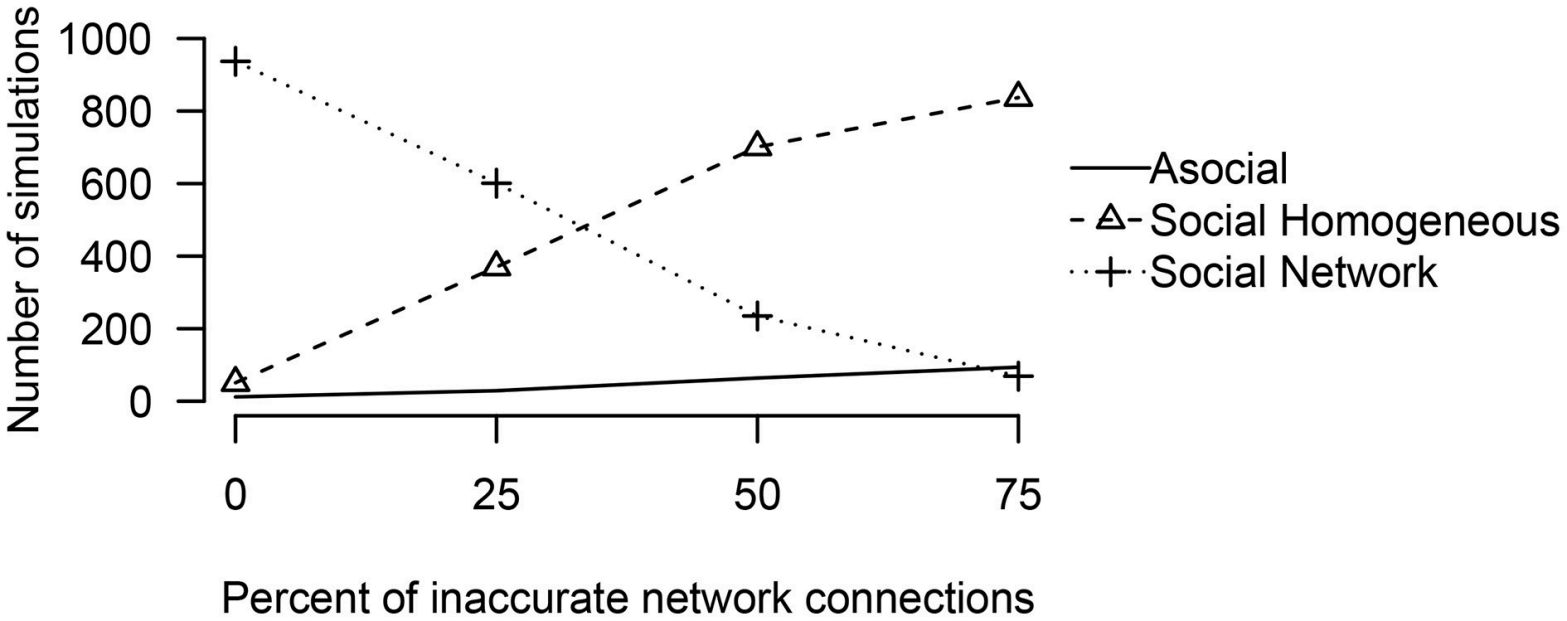
**The number of simulations either an asocial, social, or a social model with a non-trivial social network, was inferred to have been the source of the given data out of 1000 total simulations, varying the percent of inaccurate network connections**.

To examine how performance may change as a function of network inaccuracy, we considered three alternative social networks, which had inaccurate values for either 25, 50, or 75% of the network connections. In all of these cases, even though the actual social network was not known, the technique was overwhelmingly able to interpret correctly that social transmission was at work (see Figure 5). When the network was accurate (25% inaccurate), the technique generally inferred that the social transmission followed the measured network. However, when the network was inaccurate (50 or 75% inaccurate), the technique inferred that social transmission followed a homogeneous network.

These results suggest that in many cases, analysis of a homogeneous network is warranted, but that if a homogeneous network is preferred over a non-homogeneous network it does not imply that social transmission was equal, but may simply imply that the measured non-homogeneous network was substantially different from the true network of associations.

### 5.4. Distinguishing alternative models of social learning

**Figure 6:**
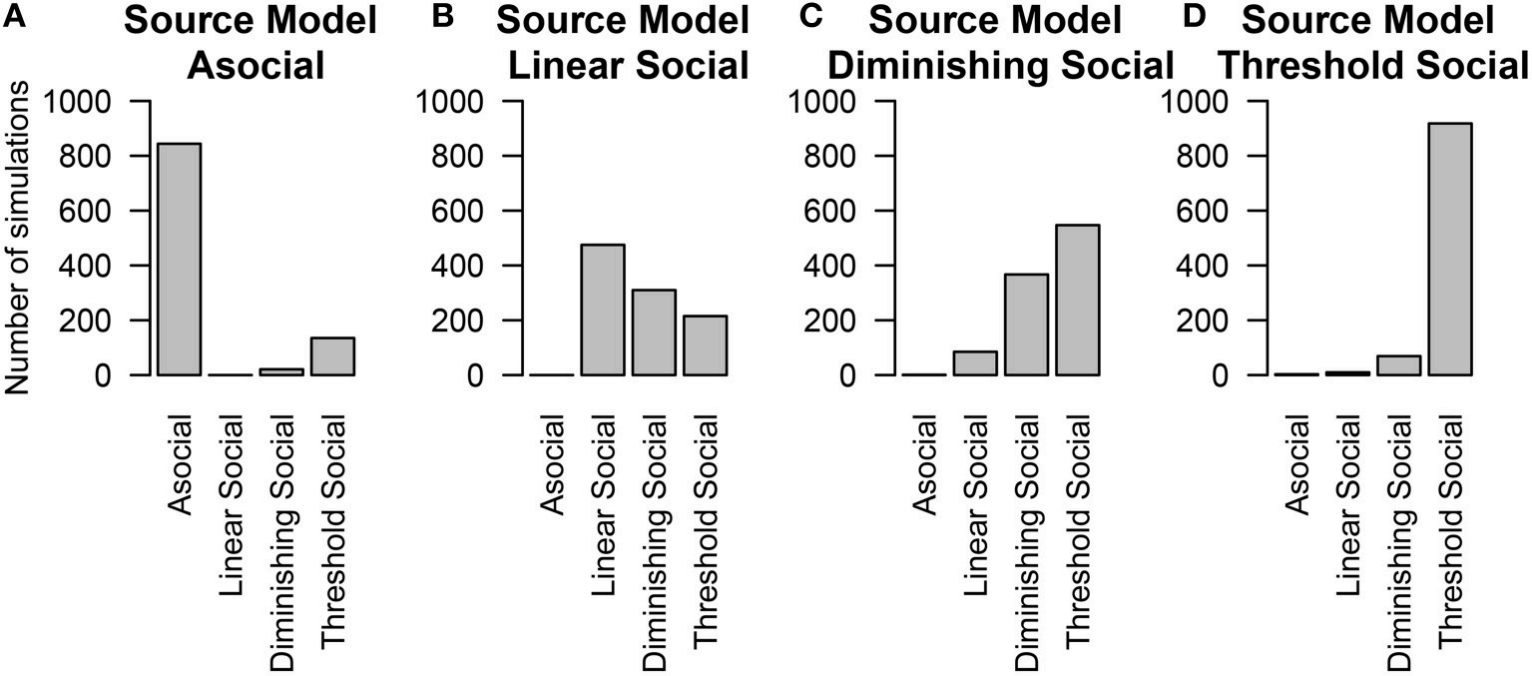
**The number of simulations each model was inferred to have been the source of the given data out of 1000 total simulations**. The data was either generated from different models of social learning, either through an **(A)** asocial model, **(B)** linear social learning model, **(C)** diminishing returns social learning model, or **(D)** a threshold social learning model. Details of each model are provided in Table 1.

The last thing we tested was whether or not we could distinguish between alternative models of social learning. In these simulations, individuals had both individual and social-level random effects and social information was transmitted across a homogeneous network. We found that in general the technique could correctly recover the true underlying model at above-chance levels (except in the case of the Diminishing Returns model, Figure 6C), and had very good performance in determining whether or not a model was asocial, or social (Figure 6).

The above findings make it clear that when the true social transmission model is included in model selection, NBDA is successful in ruling out a model of purely asocial learning. However, it is more realistic that the model of social transmission will be mis-specified in some way, i.e., at best our model with be a good approximation of the social transmission process. In most cases the linear model is assumed.

These results show that even if the underlying model was not linear, the technique could still detect the influence of social information, at least some of the time. The success rate was much higher when the true model was closer to linear, than when it was not. Furthermore, in such cases, the estimated learning parameter was generally lower than the true value of the parameter. These results suggest that if we suspect a linear model to be a poor approximation, then we should still trust the inference that social transmission is occurring. However, we should take estimates of the strength of social transmission to be conservative and understand that a negative result for social transmission may indicate that our social transmission model does not approximate the underlying process very well.

**Figure 7:**
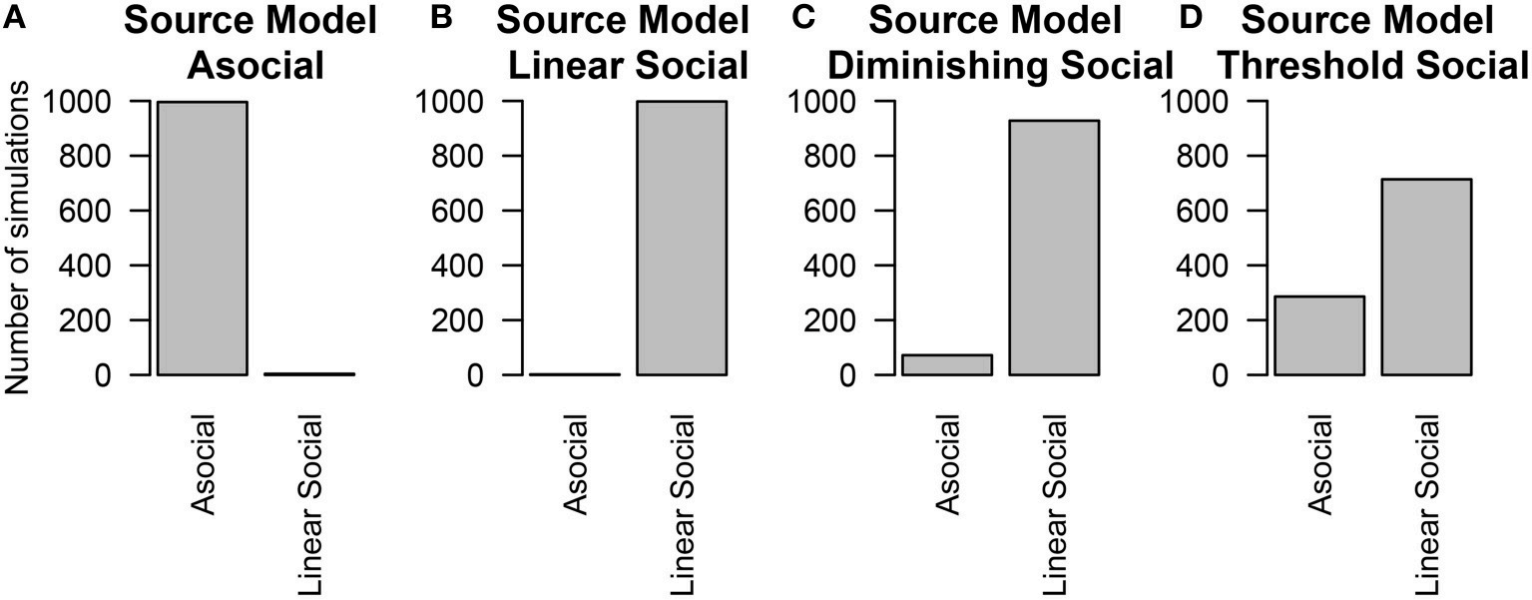
**The number of simulations each model was inferred to have been the source of the given data out of 1000 total simulations**. Only the asocial model or the linear social learning model were considered. The data was either generated from either an **(A)** asocial model, **(B)** linear social learning model, **(C)** diminishing returns social learning model, or **(D)** a threshold social learning model.

To estimate whether considering a linear model alone is sufficient for inferring the presence or absence of social information, we re-analyzed these simulations considering only the Asocial and Linear social learning models. Even if the underlying model was not linear, the technique could still detect the influence of social information, however the social learning parameter was generally lower than the true value of the parameter. Figure 6 gives the results when all four models are considered. In contrast, Figure 7 gives the results when our choice of models were restricted to an asocial and a linear social model.

Performance was worse when the influence of social information was smallest; particularly in the case of the threshold model, which will be behaviorally similar to the asocial learning model. In contrast, performance was best in the linear social learning model, where the vast majority of the time, we was able to correctly infer the correct underlying model.

## 6. General discussion

In this paper we have built on a growing literature using NBDA to infer under what conditions social information underlies the spread of novel behaviors. We present a unified framework for NBDA in the context of Time to Acquisition Diffusion Analysis (TADA). We analyzed the performance of NBDA in this context on a series of simulated experiments and found that NBDA was robust to inferring the presence of social information in most contexts, could infer the influence of random effects, and in at least some cases, can distinguish between different patterns of social learning. We also find that NBDA is robust to errors in measuring the social network behaviors diffuse across, although in some cases if a network is poorly estimated the technique will infer that behaviors diffused across a homogeneous network. In this paper we solely analyzed simulated data, for a case study for applying this methodology to experimental data see Whalen et al., (under review), or Whalen (2016).

These findings offer new insights into our ability to infer when social transmission, as opposed to asocial learning, can account for the spread of a novel behavior. However, these findings are not without their own caveats. As part of our simulation study, we found that although we could recover the correct parameter values for population-wide effects, we often underestimated the influence of random effects. Our performance in determining the presence of external correlates for learning was better, but only when the influence of these correlates was strong. We also found that we had a high error rate in determining which model of social learning was used to generate the data. Our overall accuracy of determining if social learning was used was high, but our accuracy in estimating how it was used was much lower.

These findings lead us to suggest three new recommendations for the use of NBDA. (1) When fitting models to data where multiple diffusions are run on the same individuals, researchers should fit models with random effects to account for repeated observations on individuals, but expect the estimated magnitudes of the random effects to differ from the true underlying values. (2) Individual-level effects on learning are able to be inferred, but researchers should rely on credible intervals as providing the plausible magnitude of the effect, rather than using model selection to infer its presence/absence. (3) Researchers should focus on a single, likely model of social learning to detect the presence of social information. Our results suggest the standard NBDA model used thus far is robust to fairly major departures from the assumptions of linearity, so we suggest this is used in the absence of any reason to prefer a different model. If the use of social information is well established for the task, more detailed models of learning can be used, although a large amount of data may be required to determine the underlying shape of the model.

Unlike early versions of NBDA (Franz and Nunn, 2009; Hoppitt et al., 2010), in this paper we present NBDA in the context of a Bayesian methodology, which allows us easily to include the influence of random effects. However, while most previous studies using Bayesian NBDA have used Reversible Jump Markov Chain Monte Carlo methods to perform model selection (Boogert et al., 2014; Nightingale et al., 2014), we suggest using an information criteria approach. While RJMCMC, and other methods of approximating a posterior over models are an alternative way of performing model selection, these methods are computationally difficult to perform especially when models have a large number of parameters, as occurs when including individual-level random effects. The influence of random effects is a driving factor for the development and use of a Bayesian version of NBDA, since including random effects allows us to model cases where the same individuals take part in multiple diffusion, and reduces the impact of random effects (particularly in asocial learning) as a confound in inferring the presence of social information. Consequently, we believe that using a WAIC-based approach provides an ideal tradeoff between tractability in performing the analysis, and the advantages conferred by inclusion of random effects. We recommend using Bayesian NBDA with WAIC use for examining future diffusion experiments, and believe it can give us insights into how other animals learn, and how that might influence the development of animal and human culture.

## Author contributions

AW, WH designed, carried out, and drafted this contribution together. The simulations were coded and executed by AW.

## Funding

AW was supported by John Templeton Foundation Grant #40128, and WH by BBSRC grant (BB/I007997/1).

### Conflict of interest statement

The authors declare that the research was conducted in the absence of any commercial or financial relationships that could be construed as a potential conflict of interest.
